# Effects of aging on timing of hibernation and reproduction

**DOI:** 10.1038/s41598-018-32311-7

**Published:** 2018-09-17

**Authors:** Claudia Bieber, Christopher Turbill, Thomas Ruf

**Affiliations:** 1 Department of Integrative Biology and Evolution, Research Institute of Wildlife Ecology, University of Veterinary Medicine, Vienna, Savoyenstraße 1, 1160 Vienna, Austria; 2 Hawkesbury Institute for the Environment, Western Sydney University, Locked Bag 1797, Penrith, NSW 2751 Australia

**Keywords:** Ecophysiology, Ageing

## Abstract

Small hibernators are long-lived for their size because seasonal dormancy greatly reduces predation risk. Thus, within a year, hibernators switch between states of contrasting mortality risk (active season versus hibernation), making them interesting species for testing the predictions of life-history theory. Accordingly, we hypothesized that, with advancing age and hence diminishing reproductive potential, hibernators should increasingly accept the higher predation risk associated with activity to increase the likelihood of current reproductive success. For edible dormice (*Glis glis*) we show that age strongly affects hibernation/activity patterns, and that this occurs via two pathways: (*i*) with increasing age, dormice are more likely to reproduce, which delays the onset of hibernation, and (*ii*) age directly advances emergence from hibernation in spring. We conclude that hibernation has to be viewed not merely as an energy saving strategy under harsh climatic conditions, but as an age-affected life-history trait that is flexibly used to maximize fitness.

## Introduction

The pace of mammalian life-histories correlates with body size, such that smaller species typically have shorter lifespans and higher investment in growth and reproduction than larger species^1^. However, certain traits that reduce mortality risk, such as flight, arboreality or eusociality, can lead to the evolution of relatively slow life histories even in small mammals^2–4^. Another trait that enhances survival probability, especially in small mammals, is hibernation^2,5,6^. In fact, for small hibernators, the monthly probability of mortality is five times higher during the active season than during the hibernation season^6^. Hibernators are often well hidden in underground burrows, motionless, cold, and with minimal release of odour^7^, which increases their average monthly survival to nearly 100%^6,8–10^. In accordance with their higher annual survival rates, small hibernators reproduce at a slow rate with long generation times, and hence exhibit life histories comparable to mammals of much larger size, e.g., small ungulates^6,11^. However, there remains a striking difference: whereas non-hibernating species typically have to endure environmental conditions and predation pressure above ground year-round, small hibernators can choose when to escape into their secure hibernacula and possibly even slow down physiological aging^6,12,13^.

It might seem that for any species and habitat, there is, due to intrinsic and environmentally imposed energetic limits, a fixed optimal time period during which it is necessary to hibernate. Moreover, from a physiological point of view one would expect that hibernation, (i.e. the expression of torpor) is employed only in response to severe energy shortage from a lack of food or fat reserves and is minimised to avoid negative effects of torpor^14–18^. Yet, evidence suggests that seasonal timing of hibernation in fact is reasonably flexible and not tightly constrained by energetic limitations. For instance, female Columbian ground squirrels (*Urocitellus columbianus*) show strong within-individual phenotypic plasticity in the date of emergence^19^. In the edible dormouse, *Glis glis*, large fat reserves prior to hibernation lead to a shortening of torpor bouts but do not affect the total hibernation duration or the date of emergence^20^. Thus, the timing of hibernation is neither completely explained by energetic constraints nor solely determined by environmental conditions, but seems a plastic behavioural and life history trait that can be adjusted by individuals to maximise their fitness.

Timing of hibernation is closely linked to reproduction^21,22^, which further increases its evolutionary potential, especially since variation in the day of emergence shows some heritability in both sexes^23^. Since hibernation or torpor is associated with gonadal atrophy and prevents reproduction in many species from seasonal climates^24–29^, the date of emergence and return to normothermy is a critical time point affecting the onset of reproduction. Several studies show that an earlier emergence in spring leads to higher reproductive success^30–33^. Further, in alpine marmots (*Marmota marmota*), edible dormice, and in garden dormice (*Eliomys quercinus*) an earlier birth date leads to an increased body mass prior to hibernation in juveniles^34–36^, which increases their probability to survive the first hibernation.

Whereas an earlier emergence might be advantageous for reproduction, it is, on the other hand, associated with the risk of exposure to harsh environmental conditions with low food availability and an increased risk of predation. In small non-hibernating rodents, as well as in hibernating species (e.g., edible and common dormice, *Muscardinuns avellanarius*), mortality risk is highest during the early active season, probably due to maximum predation pressure at this time of the year^8,9,37^. The time of emergence from hibernation should be optimised, therefore, to resolve a strong trade-off between the negative effects of hibernation on reproduction and its positive impact on survival.

Life history theory predicts older animals to invest more into reproduction, despite the associated survival cost, because of decreasing chances for future reproduction^38^. However, the empirical evidence for this prediction is ambiguous and measures of reproductive performance among mammals, such as litter size or birth weight, often decline toward the end of the lifespan^39^.

Individual variation in the timing of hibernation has been rarely examined^9,19,23,40,41^. To our knowledge, there are no long-term studies addressing the effect of age (i.e., age in years instead of age-classes) on timing of hibernation and current reproductive success or survival. Small hibernators could provide an exceptional model to test this theory in mammals. Indeed, in a preliminary study on a data set limited to individuals of ages 1 to 4, we detected an impact of age on hibernation patterns^40^. This led us to follow this avenue by evaluating a long-term data set, including further important traits like reproduction and individual quality in our modelling procedure.

Disentangling the factors influencing hibernation patterns over the lifetime of free-living small mammals is extremely difficult, due to dispersal and extrinsic mortality^42^ as well as unknown exact hibernation onset and termination dates in capture-recapture studies. To overcome these issues, we analysed a database containing of 289 hibernation seasons from 75 individuals living and reproducing under semi-natural conditions in large outdoor-enclosures between 2004–2014. We hypothesised that, with increasing age, dormice should (*i*) emerge earlier and shorten the hibernation duration, and (*ii*) increase their investment into reproduction. However, we also considered (*iii*) the alternative possibility of senescence (e.g., reproductive senescence) in our population, and no effect of age on the timing of hibernation.

## Results

### Effects of age on reproduction and onset of hibernation

**Figure 1:**
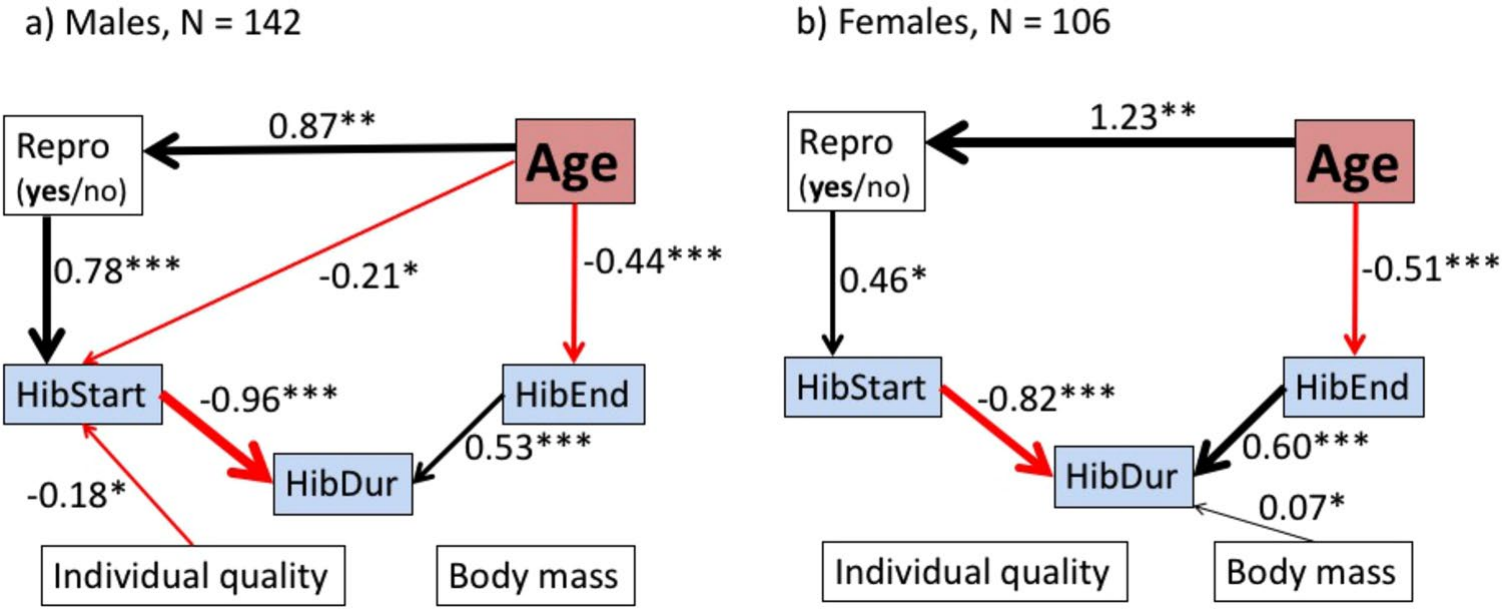
Path analysis of effects of age on reproduction on hibernation in *Glis glis*. The path analysis based on piecewise structured equation models for (**a**) males and (**b**) females. Boxes represent measured variables (red = age, blue = variables related to hibernation) and can appear as response variables in one path or as a predictor variable in another. For clarity, the binomial variable “sexually active” (Repro: yes/no) is also printed in one box. The impact of age on HibStart (hibernation onset) was partly indirect and caused by sexual activity. Arrows represent unidirectional relationships among variables (black = positive effect, red = negative effect). Shown are all included fixed variables, but only significant (P < 0.05) effects are represented by an arrow (for more details, i.e. non-significant effects see Supplementary information S1). The thickness of each significant path arrow has been scaled on the magnitude of the standardized regression coefficient. Asterisks indicate the significance level: *P < 0.05, **P < 0.01, ***P < 0.001, marginal and conditional R^2^ are given in the text (see also Supplementary information S2). HibStart = onset of hibernation (day of year), HibDur = hibernation duration (**d**), HibEnd = end of hibernation (day of year), Age = log (age) in years, repro = sexually active yes/no (for details see Material and Methods), Individual quality = lifespan of the individual (years), Body mass was measured in g prior to hibernation.

**Figure 2:**
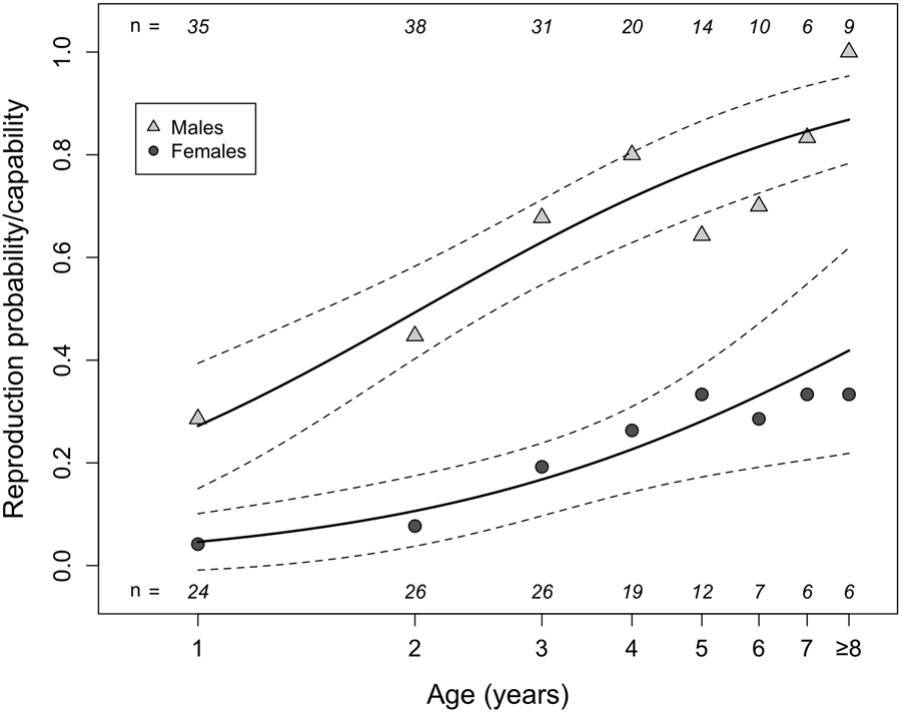
Effect of age on the probability to reproduce in dormice (females: probability to give birth to a litter, males: probability to develop sexually competent testes in the current active season). Please note that, to ease visualisation, proportions (symbols), predictions (lines) and 95% confidence intervals (dashed lines) are shown, and all ages at or above 8 years were pooled due to small sample sizes. All statistical evaluations were based on individual data. For comparison the reproductive probability (females) or capability (males) as a function of age on a linear scale are shown in Fig. S4.1.

Our path analyses revealed, due to high standardized regression coefficients, a strong positive impact of age on reproduction in both males and females (Fig. 1, Supplementary information S1). The prediction from our model showed an increasing probability to invest into reproduction in males, from 0.31 at age 1 to 0.85 at age 10, and in females from 0.03 at age 1 to 0.64 at age 10 (for details see Supplementary information, Table S2.1). Since a logarithmic scaling of the predictor age fitted our data best, there was a non-linear increase in reproduction probability (Fig. 2). The mixed model describing the reproductive probability best included the predictors log (age), age at death (i.e., individual quality) as well as animal ID and diet as random effects (same model for both sexes). The marginal and conditional R^2^ from this model were 0.19 and 0.57 in males, and 0.21 and 0.41 in females, respectively (for details see Material and Methods and Supplementary material S1).

**Figure 3:**
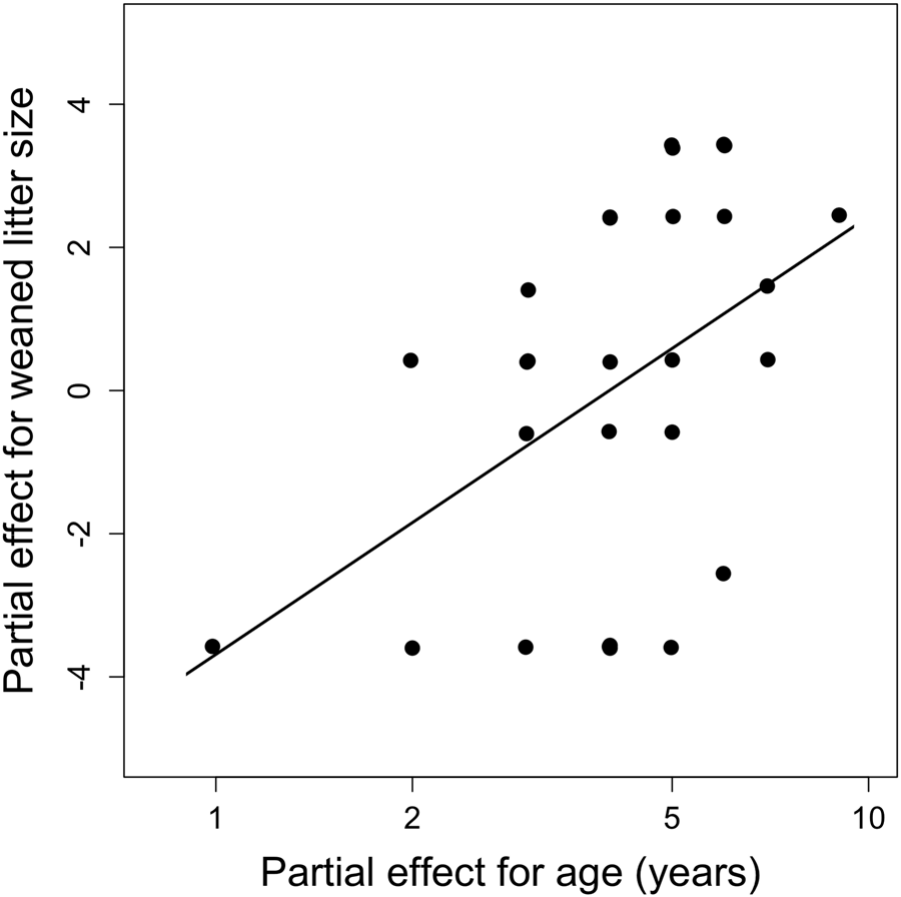
Partial effect of log (age) on weaned litter size in female edible dormice. Please note that these results were obtained from a subsample of our data set, for which the exact number of offspring of each female could be determined (N = 28 litters). For comparison, the partial regression plot of linear effects of age on weaned litter size is shown in Fig. S4.2.

The number of weaned juveniles (reduced data set, see Material and Methods) increased with log (age) (Fig. 3). The best model only included log (age) and age at death as fixed predictors besides animal ID and diet as random effects (marginal R^2^ = 0.22, conditional R^2^ = 0.33). There was no indication for an effect of body mass in spring (ΔAICc = 8.4) on reproductive output in females.

Compared with years without reproduction, the occurrence of reproduction delayed the onset of hibernation by 32.9 ± 7.7 SE days in males and by 15.7 ± 7.9 SE days in females. Thus, we observed a strong indirect effect of age, via reproduction, on the onset of hibernation in both sexes (Fig. 1). There was a direct, negative impact of age on the onset of hibernation in males only (Fig. 1a). Older males entered hibernation earlier, independent of their investment into reproduction (at age 10, onset 30.73 ± 14.84 SE days earlier than at age 1, shifting from the beginning of September to the beginning of August, Table S2.1). The impact of individual quality (i.e., age at death) of an animal was significant only among males and affected negatively only the onset of hibernation (−3.04 ± 1.41 SE days, Fig. 1a). The effects of age and longevity were, comparing standardized regression coefficients, much weaker than the effect of reproduction on the onset of hibernation (Fig. 1). The mixed models explaining the onset of hibernation in our path analysis included the predictors reproductive activity, age at death, log (age), and body mass prior to hibernation. The random effects were year, diet and animal ID. For the models describing the onset of hibernation the marginal and conditional R^2^ were 0.18 and 0.99 for males and 0.07 and 0.99 for females, respectively.

### Hibernation duration

Males hibernated on average for 8.4 months, females for 9 months. There was no impact of hibernation onset on the end of hibernation (Fig. 1). Based on the standardized coefficients the onset of hibernation had a stronger effect on hibernation duration than on the end of hibernation (Fig. 1). In females only, we observed a weak but significant positive effect of body mass on hibernation duration (0.1 days ±0.04 SD per gram body mass), reflected by a low standardized regression coefficient. Both models included the predictors reproductive activity, onset and end of hibernation and the random effect year, diet and animal ID. Marginal and conditional R^2^ for the hibernation duration models were 0.99 and 1.00 in males, and 0.89 and 0.99 in females, respectively.

### End of hibernation

**Figure 4:**
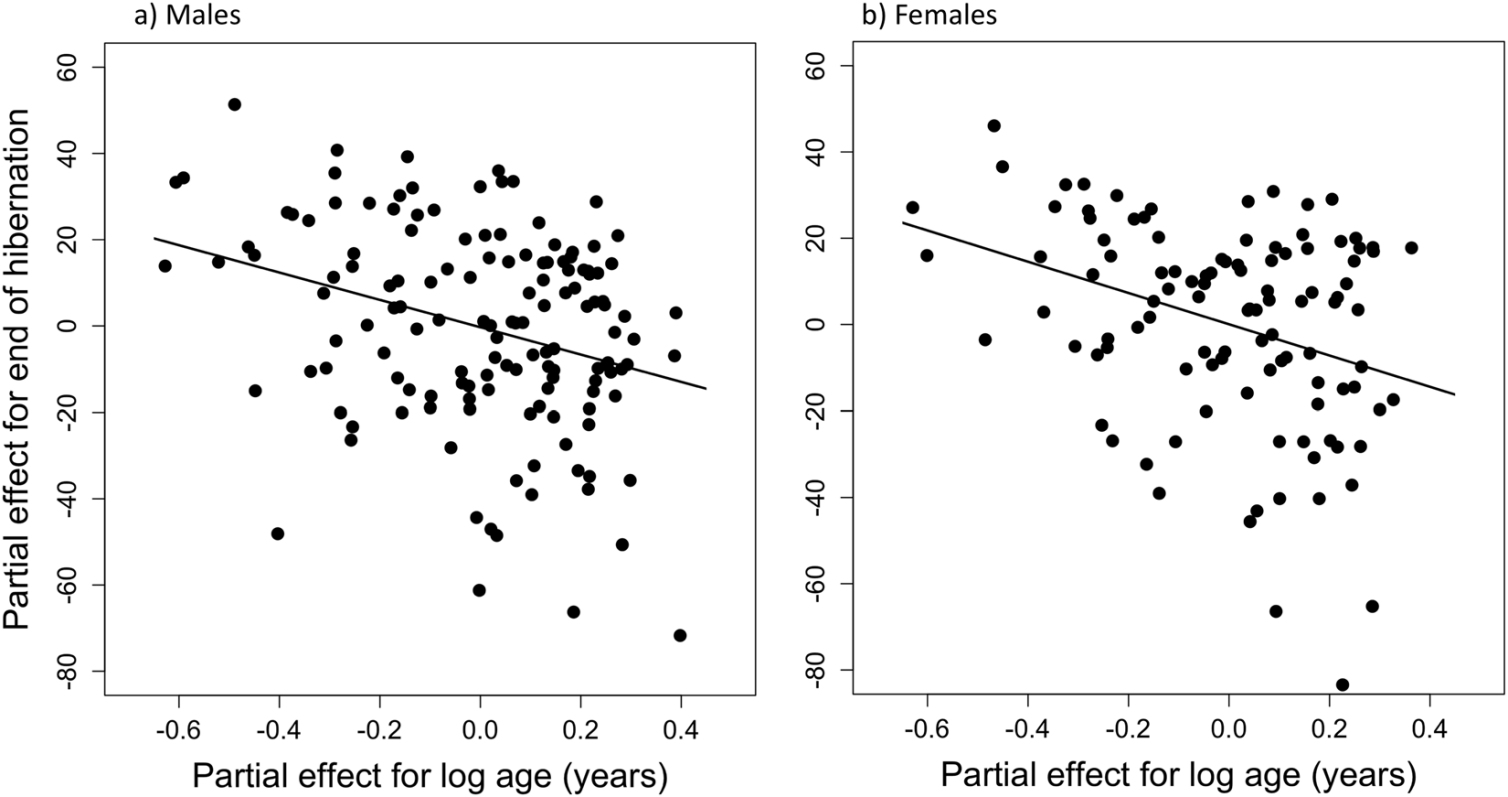
Partial effects of age (log) measured in years on the end of hibernation in males and females. For details of the path analysis see text and Material and methods. For comparison, the partial regression plot of linear effects of age on the time of emergence from hibernation is shown in Fig. S4.3 (males) and Fig. S4.4 (females).

Age had a strong impact on the termination of hibernation in both sexes (Figs 1 and 4). In males, the model-predicted day of emergence shifted from end of May to mid-April as age increased from 1 to 10 years, corresponding to a phase advance by 35.94 ± 9.00 SE days. In females, this change was even greater i.e., 45.04 ± 10.46 SE days (shifting from the middle of June to the beginning of May). Both models (males and females) explaining the variation in the termination of hibernation included the predictors log (age), age at death, reproductive activity, and body mass prior to hibernation. Random effects were year, diet and animal ID. The marginal and conditional R^2^ for the end of hibernation model were 0.18 and 0.94 in males, and 0.24 and 0.94 in females.

### Survival

**Figure 5:**
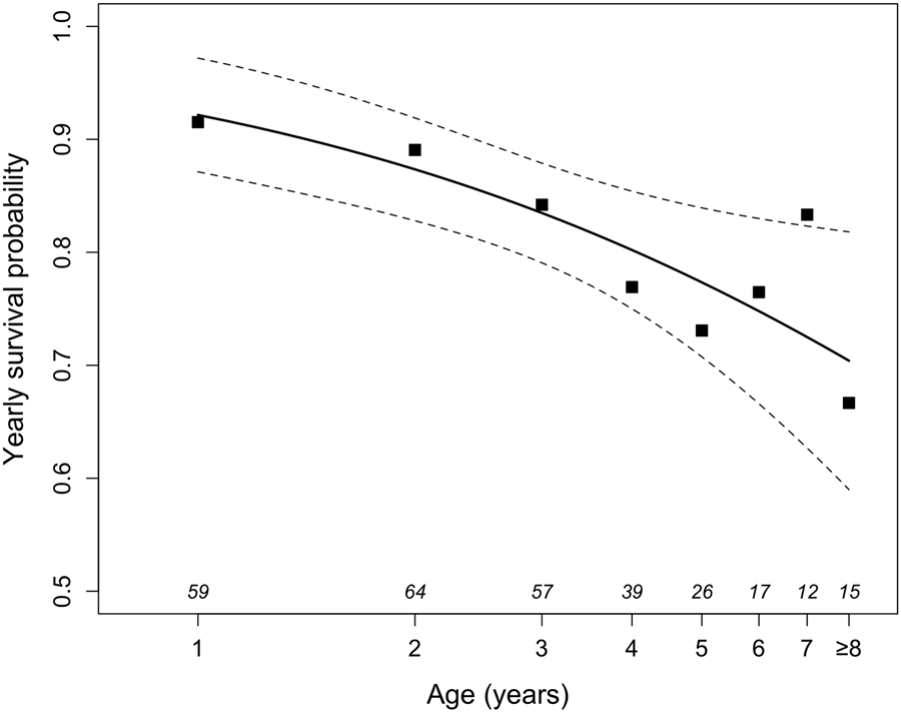
Age dependent yearly survival probabilities in enclosure housed edible dormice. Since there was no significant sex effect on survival, data for males and females were pooled. To ease visualisation, proportions (symbols), predictions (line), and 95% confidence intervals (dashed lines) are shown, and ages at or above 8 years are pooled due to small sample sizes. All statistical evaluations were based on individual data. The number of animals is given at the bottom of the figure. For comparison, effects of age (on a linear scale) on yearly survival in dormice kept in outdoor enclosures are shown in Fig. S4.5.

Survival probability decreased with increasing age (ΔAICc = 5.5), without a notable sex difference (Fig. 5). The predicted yearly survival probability decreased from 0.91 ± 0.01 SE at age 1 to 0.65 ± 0.03 SE for animals aged 8 or older. Note that these high survival rates were determined under conditions excluding predation. In addition to the random effects animal ID and year, the best model for survival rates included log (age) as well as an interaction between sex and reproduction (yes/no). This was because among females, survival was much lower in reproductive than in non-reproductive years (0.65 ± 0.03 SE vs. 0.90 ± 0.01 SE, but there was no effect of reproduction on survival among males (0.82 ± 0.01 SE vs. 0.83 ± 0.01 SE, respectively). The second-best model (ΔAICc = 1.2) additionally contained an interaction between age and reproduction: among younger females (<5 years) survival decreased only moderately from 0.91 ± 0.01 SE in non-reproductive to 0.74 ± 0.03 SE in reproductive years (−19%), whereas among older females (≥5 years) reproduction led to a more severe decline in survival probability from 0.84 ± 0.01 SE to 0.54 ± 0.03 SE (−36%).

## Discussion

**Figure 6:**
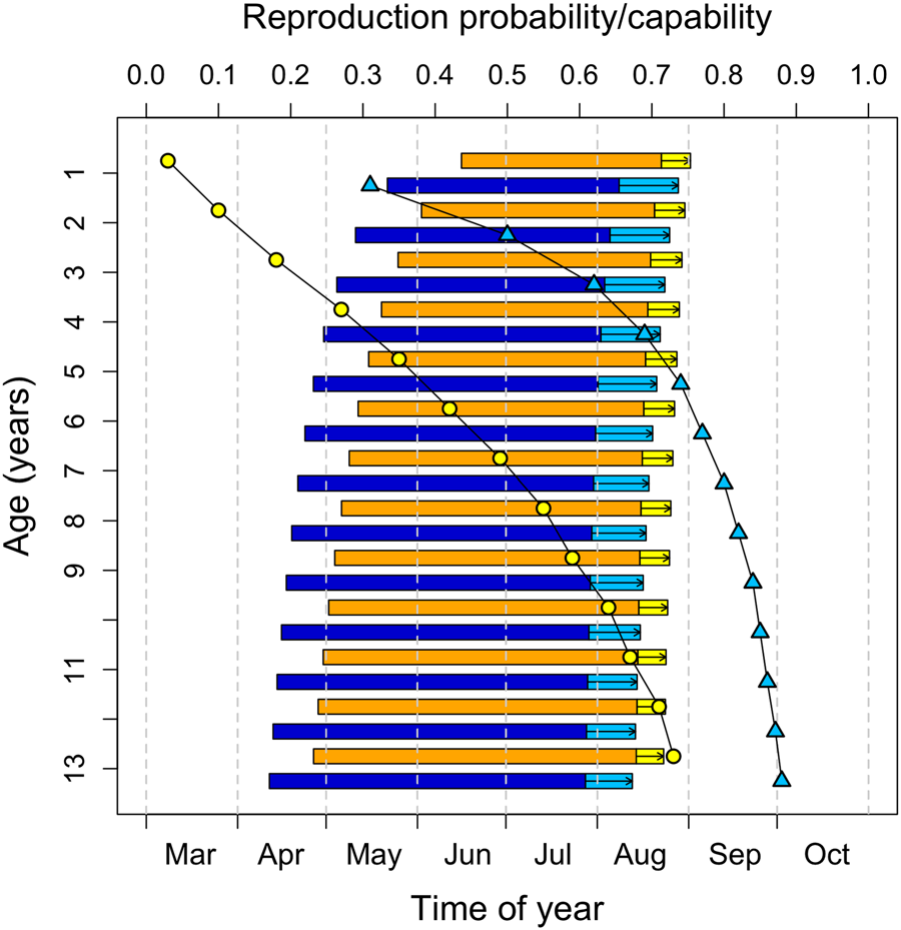
Graphical summary of effects of age and reproduction on the timing of hibernation and activity. As age increases both emergence from hibernation and hibernation onset occur earlier in the year, leading to a phase advancement of the active season (horizontal bars; blue: males; orange: females). Also, the proportion of sexually active males with large testes (blue triangles) and of reproducing females (yellow circles) increases with age. Investment into reproduction leads to a delay in hibernation onset in both sexes (light blue and yellow bars with arrows). Shown are model predictions for non-reproducing dormice, as well as predictions based on the observed fraction of reproductively competent/active animals in each age class.

Our path analysis revealed that dormice emerged earlier from hibernation with increasing age, and that this was a direct effect. The impact of age on the onset of hibernation, on the other hand, was mediated via reproduction. The probability of reproduction increased with increasing age, and reproductively active animals of both sexes delayed the onset of hibernation significantly. As hypothesized, the net result of the combined age effects on hibernation was a decrease in hibernation duration in older animals (Fig. 6). We argue that this phase-advancing of hibernation is an age-dependent life-history trait that serves to maximise fitness.

Before considering such an argument we must be convinced, however, that age-dependent timing of hibernation is due neither to a cohort effect, caused by differences in individual quality, nor to an acquired behaviour triggered by the animals receiving food supplies and being protected from predation. We can eliminate the first possibility of persistent differences between individuals that died young and those with high longevity because we adjusted all analyses for age at death, which had only a weak impact. Further, high body mass did not lead to an earlier emergence from hibernation, which is in line with earlier findings^20^.

Is it possible then, that captive animals become accustomed to continuous food supply, emerge early by chance and will continue to do so in the subsequent year? This type of learning might be falsely interpreted as an age effect. However, our dormice experienced substantially varying food quality, including even fat-reduced diets (dry cat food/apple), in a random sequence unrelated to their age. Similarly, we consider it unlikely that dormice learned to emerge early into a safe, predator-free environment. Firstly, foxes, martens, and owls were present around the enclosures, which were situated within a large natural woodland, and these predators could be perceived by the dormice via sight, sound, and odour. We observed during the whole study that the animals in our population react when humans approach the enclosures during night-time (they stop whistling, a common vocal behaviour especially in the mating season), which indicates that the animals are continuously alert and cautious about their environment. Secondly, any learning component would not explain, by itself, why the animals should terminate hibernation progressively earlier every year. It is true that dormice, like other hibernators, avoid negative effects of torpor, such as oxidative stress during periodic arousals, if they have large energy reserves^17,18,20,43^. However, in edible dormice this avoidance is manifested by longer periods spent euthermic during hibernation, and by regulation of higher body temperatures in torpor, not by shortening the hibernation season^20^.

For these reasons, we consider the effect of age on the timing of hibernation a genuine life-history trait. Arguably, it is intrinsically linked to another observed effect of age, namely that on reproduction (Figs 2, 6). Notably, the lack of a decline in reproductive activity with increasing age, as observed here, also occurs in free-living females^12^. Among free-living dormice the probability to reproduce stayed low within the first years of life, sharply increased at age 4–5 and remained continually high up to the oldest age observed (8 years)^12^. According to life-history theory, long-lived species should, with advancing age, increasingly favour investment into reproduction relative to investment into survival^38,44,45^. Indeed, our findings in the long-lived edible dormice (up to 13 years^46^), support the ‘terminal allocation hypothesis’, which predicts an increase in reproductive effort in late-life^47,48^. However, the increasing reproductive activity together with the increasing litter size in dormice, as suggested by the present study, contrasts with general findings (and therefore refutes our alternative reproductive senescence hypothesis) in which reproductive output increases early and declines late in life^39,49–51^. The absence of a recruitment senescence (i.e., declining numbers of weaned young per year), was also found in other hibernating species such as yellow bellied marmots (*Marmota flaviventris*) and Columbian ground squirrels but often occurs in large mammals^52^. A prerequisite for assuming terminal allocation are progressively diminished chances for future reproduction. This condition is met by our finding of a decline in survival probability with increasing age (i.e., actuarial senescence; Fig. 5).

The major reason why dormice should be particularly prone to adopt a terminal allocation strategy is their adaptation to pulsed resources. Edible dormice reproduce only in mast years when at least some seeding beech or oak trees are present, because juveniles require high-caloric seed for pre-hibernation fattening^11,53,54^. Thus, as an adaptation to intermittent tree masting, dormice have evolved a strategy to “sit-tight” without reproducing in non-mast years, awaiting years of sufficient food availability for investment into reproduction^53^ – a pattern expected by life-history theory for extremely variable environmental conditions^55^. Depending on their actual home-range, food may be sufficient for reproduction in intermediate mast years, when only a fraction of trees produces seeds. On average, female dormice reproduce approximately every other year^56^, which should allow a sufficient frequency of opportunities to lead to a terminal investment strategy. The selective advantage of a shift towards reproduction in the current year with increasing age is also underlined by our observation of a strong trade-off between reproduction and yearly survival among old females. Surprisingly, this trade-off occurs not only in free-living dormice^53^, but was even detectable under conditions of *ad libitum* feeding in the present study, which is very unusual^57^.

There are several pathways by which reproduction affects the seasonal timing of both onset and termination of hibernation. Reproductively active females delayed the onset of hibernation, arguably due to the time required for lactation (5–6 weeks) and pre-hibernation fattening. This delaying effect was, unexpectedly, somewhat weaker in females than in males (Fig. 1). This result may be explained by the fact that we considered only whether a female gave birth but not whether the litter was raised successfully. Losing a litter will reduce energy expenditure and hence lessen the delaying effect of reproduction on the onset of hibernation in females. Males, on the other hand, are likely to be less flexible and maintain functional testes along with high levels of androgens as long as they might encounter a female in oestrus, which typically prevents hibernation onset^24^. This might be even late in the active season since females that have lost a litter during pregnancy or very early after birth can give birth to a replacement litter^58^.

While the effects of reproduction on the onset of hibernation are apparently caused by gonadal activity, lactation and energy requirements, it seems that age-effects on emergence from hibernation are associated with reproductive success in the upcoming active season. In our enclosures males emerged on average 2–4 weeks prior to females, reflecting a pattern occurring also in natural populations^54^. Age caused both sexes to advance emergence from hibernation by more than one month during their lifetime (from age 1 to 10). The timing of seasonal activity onset apparently involves a trade-off. An early emergence typically coincides with times of low food availability, cold temperatures and high predation pressure, since owls, the main predators of dormice, are raising their young and show maximum foraging activity during early spring^59^. Still, an age-dependent shift towards earlier emergence could have at least two benefits. Firstly, an early emergence is known to increase reproductive success in other hibernators^30,31,33^ because early activity (*i*) increases the number of potential mating opportunities for males and (*ii*) allows females and their young to maximise the time for pre-hibernation fattening. For instance, in yellow-bellied marmots a progressively earlier emergence from hibernation, due to climate change, has led to earlier weaning of young, and drastic increases in population size^22^. Secondly, animals emerging early at a site are more likely to occupy high-quality feeding territories^60,61^, which can substantially differ and affect reproduction in dormice^56,62^. This might be especially pronounced in intermediate mast years, with a lower proportion of seeding trees^63^.

Costs and benefits of the state of torpor contribute to a highly flexible use of hibernation among and within species. For instance, within the same species there is variation among populations, e.g., along latitudinal clines^64,65^ or even in response to subtle differences in local climate^66^. Further, within populations, individuals adjust their torpor use and patterns to climatic conditions of individual hibernacula^67–69^, or short-term fluctuations of temperature or food availability^17,70^. High energy reserves (i.e., body fat or food stores) can decrease the use of torpor^18^ or affect the timing of hibernation^71^. On the other hand, if energy reserves are high, the use of torpor or the duration of hibernation can be extended to avoid predation^72,73^. Our present results add, however, a new dimension to this flexibility as they show that individuals adjust torpor timing not only to energy availability or external conditions, but also to their changing endogenous state, particularly age and hence residual reproductive value. Currently it is unknown by which physiological mechanisms age affects the timing of hibernation. Possibly cellular aging processes may affect the molecular mechanisms that drive the circannual clock, which governs dormancy/activity cycles in hibernators, including edible dormice^74–76^.

Irrespective of the proximate mechanisms involved, there are three reasons to think that aging affects the timing of dormancy in hibernators in general, not just in edible dormice. First, as outlined above, hibernators have high longevities compared with similar-sized non-hibernators^6^, which is a prerequisite for relevant aging effects. Second, the trade-off between the costs and benefits of early emergence from hibernation, i.e., exposure to predators and unfavourable climate versus an early start to reproduction, arguably is a general pattern. Although hibernation is an energy-saving strategy, equally important are the life-history consequences of the cryptic behavioural state, typically in closed burrows, and hence predator avoidance during hibernation. Our results demonstrate how timing of hibernation, which is an integral part of the timing of reproduction, is adjusted presumably to reflect a shift in the optimal solution for a cost/benefit trade-off over the lifespan of a hibernating mammal. As hibernation and torpor is widespread and occurs in at least 11 mammalian orders^77^, its effects on life history strategy potentially affect a substantial number of species. Further, it is plausible that changes in the timing of hibernation may affect whole ecosystems. For instance, climate change and food resources provided by humans (e.g., rubbish dumps) additively affect timing of hibernation and in consequence prey-predator interactions with severe consequences, as recently shown for black bears^78^. Thus, understanding mechanisms affecting the flexibility of hibernation and raising awareness of the impact of hibernation in ecology and conservation research seems an important issue. More generally, the age effects observed here ultimately are caused by a common challenge for animals of resolving a trade-off between reproductive opportunity and activity-dependent predation risk when environmental resources vary strongly over time or space. Thus, age-dependent switching between less active cryptic and conspicuous active behavioral states may well affect many non-hibernating species.

## Materials and Methods

### Animals, diet and climate

Edible dormice have a body length of 12–14 cm with a bushy tail of ~10 cm length, and are distributed in Europe. We kept dormice in our colony (founded in 1996) in mixed age and sex groups year-round in outdoor enclosures (ca. 6 × 4 × 3.5 m) during the study period (2004–2014). The animals were held in stable groups (9–12 animals per enclosure, juveniles not considered), but, to avoid inbreeding, juveniles were moved between the enclosures. The fenced enclosures were located at the edge of a deciduous forest and, while the animals were not exposed to predation, they were, however, able to perceive the presence of predators (i.e., calls from owls, straying foxes and martens) next to the enclosures. Each enclosure covered ~24 m^2^ ground with soil at the bottom, enclosed by a concrete foundation (0.8 m depth), where the animals could dig their hibernacula. The ground in all enclosures was covered with grass and 1–2 bushes of European elder (*Sambucus nigra*), inflorescences of the bushes were removed before flowering to avoid ripening of fruits and thus the availability of additional food resources. The enclosures were located 362 m a.s.l. in Vienna, Austria (48°13′ N; 16°16′ E). We marked all animals individually with passive integrated transponders (Trovan®, Backhome BioTec®, Tierchip Dasmann®) and captured them in their nest-boxes (sleeping sites) once a week during the active season. The number of nest-boxes provided was one per animal (yearling or adult) or, with juveniles around, at least so many nest-boxes that one remained always unoccupied. Thus, there was no limitation of nest-boxes for the animals (i.e. breeding-site opportunities for females). During weekly checks we recorded body mass to the nearest 1.0 g. We searched enclosures carefully to assure that all active dormice were captured. Dormice in our enclosures, like the wild population in surrounding areas (Vienna woods), raised a maximum of one litter per year^58^. We recorded whether a female had a litter during weekly nest-box controls. To minimise disturbance, we counted the juveniles per litter only at weaning (5–6 weeks). The mothers were not weighed for 3 weeks after birth. However, we could not record the exact litter sizes from all females since in some cases two females raised their litters together. Thus, we used for our modelling whether a female had a litter (i.e., giving birth, visible nipples yes/no). To evaluate effects of age on litter size at weaning we used a reduced data set with known weaned litter size and age at death (n = 28 litters) and analysed these data separately. In males the length (nearest 1.0 mm) of the left testis was recorded every week. We considered males with a testis length ≥20 mm (range 5–26 mm) for at least three consecutive weeks to invest into reproduction since larger testis are known to carry more sperm in dormice^79^. The threshold of ≥20 mm was based, conservatively, on results of a field study where the length in a food supplemented group was circa 20 mm (Fietz *et al*. 2009). Only in this condition 100% of the males where sexually active. While the animals occupied the offered nest-boxes during the active season, all dormice exclusively used underground burrows, dug by the animals themselves, for solitary hibernation^5^.

Except for one animal all dormice were born in our enclosures (one female was captured as a yearling in the field). Thus, we were able to determine the exact age for each animal. Per definition, animals reached age one after termination of their first hibernation season and stayed in this age-class until termination of their second hibernation season, and so on. We collected data of 289 hibernation seasons from 75 individuals in a combination of cross-sectional and longitudinal data. Thus, we observed animals with the same age in different years (born between 2002 and 2011). Hibernation seasons were recorded between one and nine times per individual (n = 7 one season, 10 two seasons, 22 three seasons, 17 four seasons, 4 five seasons, 4 six seasons, 6 seven seasons, 4 eight seasons, 0 nine seasons, 1 ten seasons). Some animals were born late during the study period (2011) and were still alive at the end of the study. Individuals were aged between one and ten years (n = 59, 64, 57, 39, 26, 17, 12, 7, 4, 4 for each age, respectively). We obtained 126 data sets from females and 163 from males. Only complete data sets from healthy animals were used. We did not consider juvenile dormice with their first hibernation season since it is well known that juvenile dormice enter hibernation later than all other age groups^80^. We were able to obtain the total lifespan from 64 individuals (exact age of death until summer 2016). We included the variable lifespan into our evaluation (details see below) to adjust for a measure of individual quality in our data set. Thus, somewhat reduced data sets (males: n = 142, females: n = 106) were used if the variable age at death was included in the modelling procedure (see Results and Supplementary information).

Food was provided at 3–4 feeding platforms (1.5 m high) per enclosure to allow all dormice access to food. Water was provided *ad libitum*. Dormice were generally fed *ad libitum* but the energy content of the basal diet changed between the years and/or enclosures to simulate changing food conditions like observed under field conditions^54^. The animals were mainly fed with rodent chow (Altromin 7024, 1324, Altromin, Lage, Germany and ssniff^®^ Gerbil, Soest, Germany, ME (metabolisable energy) = 123–133 kJ/100 g). In some years rodent chow was supplemented by ~500 g (per week/enclosure) of sunflower seeds (husked seed: 2427 kJ/100 g) for at least 4 weeks (simulating a mast year). In 2009 and 2011 we offered a high protein diet (dry cat food (904 kJ/100 g, protein content 30%) and fresh apple (~218 kJ/100 g)) in some enclosures (i.e., mast failure year). These three feeding regimes were considered in our evaluation (three levels of the variable diet, see below). To account for different feeding regimes the variable diet was included as a random effect into the modelling procedure (see below).

### Climate

Annual mean air temperature (http://www.zamg.ac.at/: recorded at “Hohe Warte”, Vienna, Austria, 48°15′N and 16°22′E, 203 m a.s.l.) during the study period (2004–2015) ranged from 9.9 °C in 2010 to 14.4 °C in 2008 (mean 11.3 ± 0.34 °C). The mean air temperature during the hibernation period (mean for October–April) was 5.8 ± 0.34 °C. The coldest hibernation period was observed in 2005/06 (4.2 ± 2.15 °C), the warmest in 2006/07 (8.4 ± 1.39 °C). We did not observe an effect of winter temperature or a time (year) effect on the emergence of dormice during our study period (winter temperature: F_8,1_ = 0.29, P = 0.6025, year: F_8,1_ = 0.84, P = 0.3865). Thus, in contrast to other studies on hibernators^23,81,82^ we found no evidence for an effect of climate change in our data. However, to account for annual variation in hibernation patterns, we included year as a random effect in our models (see below).

### Hibernation data

We collected hibernation data over 11 years (n = 16–35 per year, between 2004–2014). We defined hibernation duration as the time between the last capture in one year until the first capture in the following year. We knew from implanted temperature recorders in a subsample of dormice (see Supplementary information S3) that dates of onset and termination of hibernation are highly correlated with their first encounter in nest-boxes (end of hibernation: t = 15.72, df = 31, P < 0.0001, r^2^ = 0.885; onset of hibernation: t = 20.22, df = 31, P < 0.0001, r^2^ = 0.927; Fig. S3.1). Thus, we defined the period of absence from nest-boxes as hibernation duration in the present study.

### Statistics

All statistical analyses were carried out using program R Version 3.3.2^83^. We performed path analyses using the piecewise structural equation approach^84,85^ (R-package piecewise SEM^86^). Path analysis tests hypothesized causal relationships. Hence, with this method we were able to identify the pathways by which hibernation patterns were affected, considering different models and response variables (onset, end, and duration of hibernation) simultaneously. To justify our model selection, we conducted direct-separation (d-sep) tests and used Fisher’s C statistic as well as Chi-squared degrees of freedom to test the model fit (i.e., missing pathways) and to compute AICc values Akaikes Information Criterion for small sample size^85–87^. We started with a full model and removed variables that did not occur in significant pathways and increased the AICc. The path analysis based on this model list constitutes hypothesis testing, and as a measure for the importance of pathways we provide standardized coefficients for significant (P < 0.05) paths. The coefficients were standardized to allow comparison between variables. We included four linear relationships in our path analysis: Three linear mixed effects models (lme models) with the response variable onset of hibernation, hibernation duration, and end of hibernation (R-package nlme^88^) and one generalized linear model (glmer model) with the binomial response variable reproduction (females: giving birth (yes/no), males: left testis length >20 mm for at least 3 weeks (yes/no), (R-package lme4^89^). Because of different measures of investment into reproduction, we evaluated males and females in separate path analyses. We used the variable age on a logarithmic scale in these models, since this resulted in a lower AICc (ΔAICc males: 8.24; ΔAICc females: 4.80). The longevity (age at death in years) was kept on a linear scale since a logarithmic scale did not decrease the AICc. Body mass in spring does not affect the decision whether to invest into reproduction or not in this species^20,54^ and omitting this variable decreased the AICc (ΔAICc males: 28.16; ΔAICc females: 17.02). To correct for possible annual effects (year), different feeding regimes (diet, see above), and individual variation (animal ID) these variables were entered as nested random effects in all lme models. In case of the glmer model (i.e., reproduction) we added the random effects diet and animal ID only, since investment into reproduction is most likely affected by diet but not by year^54^. According to Fisher’s C statistic these models fitted our data well (males: Fisher’s C = 4.35, df = 4, P = 0.361, females: Fisher’s C = 2.8, df = 4, P = 0.591) and did not show any signs of missed pathways.

From these models we recovered normal and standardised (scaled by mean and variance) regression coefficients and their associated standard errors (SE) and P-values. Further, we computed the marginal R^2^ (variance explained by fixed effects) and the conditional R^2^ (variance explained by both fixed and random effects, R-package piecewiseSEM^86^).

The number of weaned juveniles (i.e., reproductive success in females) was evaluated in a data subset (28 litters), whenever litter size at weaning could be assigned to one mother and age at death was known. We used a full lme model with body mass in spring, age (on a logarithmic scale), and individual quality (age at death), with animal ID and diet as a random effect. Visual inspection of residuals did not show a bias in their distribution. Based on AICc we selected the best model.

Yearly survival (from emergence in year t to emergence in year t + 1; yes/no) and reproduction (yes/no) was modelled using general binomial mixed models (function glmer), starting with full models that contained age and sex as fixed effects, as well as year and animal ID as random effects. For both response variables, log (age) was the better predictor than age as such. To see if survival rates were also affected by reproduction in either sex, the full survival model also included two-way interaction between fixed effects. Starting with this model we computed all possible models and their AICc.

### Ethical approval

We declare that the implantation experiments in this study comply with the current laws of Austria in which they were performed (GZ68.205/167-BrGT/2004). Feeding regimes and animal housing were discussed and approved by the ethics committee of the Veterinary University Vienna in accordance with Good Scientific Practice guidelines and national legislation.

## Electronic supplementary material

Supplementary Information

## Data Availability

The datasets generated and analysed during the current study are available at Dryad Digital Repository (after acceptance of the manuscript).
