# Author Correction: Effects of aging on timing of hibernation and reproduction

**DOI:** 10.1038/s41598-019-51736-2

**Published:** 2019-10-24

**Authors:** Claudia Bieber, Christopher Turbill, Thomas Ruf

**Affiliations:** 1 Department of Integrative Biology and Evolution, Research Institute of Wildlife Ecology, University of Veterinary Medicine, Vienna, Savoyenstraße 1, 1160 Vienna, Austria; 2 Hawkesbury Institute for the Environment, Western Sydney University, Locked Bag 1797, Penrith, NSW 2751 Australia

Correction to: *Scientific Reports* 10.1038/s41598-018-32311-7, published online 17 September 2018

This Article contains typographical errors in the Acknowledgements section.

“This project was supported by the city of Vienna, and the Austrian Science Fund (FWF, Project P20534-B17).”

should read:

“This project was supported by the city of Vienna, and the Austrian Science Fund (FWF, Project P25023).”

