# Supplementary material for: Effects of aging on timing of hibernation and reproduction
Source: Sci Rep. 2018 Sep 17;8:13881. doi: 10.1038/s41598-018-32311-7 (PMC6141465; doi:10.1038/s41598-018-32311-7)
Supplement: Supplementary file 1 — Supplementary Information [file 41598_2018_32311_MOESM1_ESM.pdf]

Supplementary Information

Effects of ageing on timing of hibernation and reproduction

Claudia Bieber[1,2], Christopher Turbill[2], Thomas Ruf[1]

[1]Research Institute of Wildlife Ecology, Department of Integrative Biology and Evolution, University of Veterinary Medicine, Vienna, Savoyenstraße 1, 1160 Vienna, Austria
[2]Hawkesbury Institute for the Environment, Western Sydney University, Locked Bag 1797, Penrith NSW 2751, Australia

E-mails: claudia.bieber@vetmeduni.ac.at, C.Turbill@westernsydney.edu.au, thomas.ruf@vetmeduni.ac.at

S1) Results path analysis (R-package piecewise SEM)

Below we provide the R-Code used to perform and retrieve results from a path analysis using R-package piecewiseSEM[1] computed from a list of models fitted using functions lme R-package nlme[2] and glmer R-package lme4[3]. Path analyses were calculated separately for males and females.

Abbreviations used:
hibstart.doy = onset of hibernation (day of year), hibdur.days = hibernation duration, hibend.doy = termination of hibernation (day of year), repro_active = females: giving birth yes/no; males: testis > 20mm for at least three weeks yes/no, log_age = age in years on a logarithmic scale, age. death = longevity (years), bm_before = body mass prior to hibernation, a.name = animal ID, diet = feeding condition (see main text).

Males:

```
modlist=list(
lme(hibstart.doy~repro_active+age.death+log_age+bm_before,random=~1|year/diet/a.name,na.action
=na.omit,data=datm),
lme(hibdur.days~repro_active+hibend.doy+hibstart.doy+age.death+log_age+bm_before,random=~1|yea
r/diet/a.name,na.action=na.omit,data=datm),
lme(hibend.doy~log_age+age.death+repro_active+bm_before,random=~1|year/diet/a.name,na.action=n
a.omit,data=datm),
glmer(repro_active~log_age+age.death+(1|a.name)+(1|diet),family=binomial,data=datm)
)

> sem.fit(modlist,datm, .progressBar = FALSE,conditional=T)

$missing.paths

missing.path                         estimate std.error df crit.value p.value
1 repro_active~bm_before+age.death+log_age
                                     -0.0034    0.0076  NA    -0.4432  0.6576

2 hibend.doy~hibstart.doy+age.death+log_age+bm_before+repro_active
                                      0.0682    0.0497 119     1.3710  0.1729




$Fisher.C
  fisher.c df p.value
1     4.35  4   0.361


$AIC

    AIC   AICc  K   n
1 72.35 94.593 34 142


> sem.model.fits (modlist)

     Class   Family     Link   n  Marginal Conditional
1      lme gaussian identity 142 0.1750271   0.9990470 (model hibstart)
2      lme gaussian identity 142 0.9945171   1.0000000 (model hibdur.days)
3      lme gaussian identity 142 0.1755294   0.9419713 (model hibend)
4 glmerMod binomial    logit 142 0.1905036   0.5721905 (model repro)

> coef.table = sem.coefs(modlist, datm)
```

```
> coef.table

       response       predictor      estimate    std.error p.value    
1  hibstart.doy repro_activeyes  32.935382406  7.732444811  0.0000 ***
2  hibstart.doy       age.death  -3.038854601  1.407956948  0.0329   *
3  hibstart.doy         log_age -30.724454509 14.842936147  0.0406   *
4  hibstart.doy       bm_before  -0.104131171  0.099344493  0.2967    
5  hibdur.days     hibstart.doy  -1.007209540  0.008064035  0.0000 ***
6  hibdur.days       hibend.doy   1.008592786  0.013907149  0.0000 ***
7  hibdur.days        age.death  -0.262526195  0.155445177  0.0939    
8  hibdur.days  repro_activeyes  -0.575418966  0.754109989  0.4470    
9  hibdur.days          log_age   0.212868851  1.416235559  0.8808    
10 hibdur.days        bm_before   0.000171043  0.009773119  0.9861    
11   hibend.doy         log_age -35.935478585  9.008333035  0.0001 ***
12   hibend.doy       bm_before   0.082843564  0.062218935  0.1856    
13   hibend.doy repro_activeyes  -3.963374964  4.643869738  0.3951    
14   hibend.doy       age.death   0.262313456  0.916064744  0.7751    
15 repro_active         log_age   3.042688245  0.945166071  0.0013  **
16 repro_active       age.death   0.179133364  0.108114756  0.0975    


> sem.coefs(modlist,datm,standardize = "scale")

       response       predictor      estimate   std.error p.value    
1  hibstart.doy repro_activeyes  0.7758052709 0.182139239  0.0000 ***
2  hibstart.doy       age.death -0.1826258057 0.084612973  0.0329   *
3  hibstart.doy         log_age -0.2076733158 0.100326436  0.0406   *
4  hibstart.doy       bm_before -0.0769855499 0.073448294  0.2967    
5  hibdur.days     hibstart.doy -0.9561310266 0.007655084  0.0000 ***
6  hibdur.days       hibend.doy  0.5261709102 0.007255195  0.0000 ***
7  hibdur.days        age.death -0.0149766956 0.008867896  0.0939    
8  hibdur.days  repro_activeyes -0.0128667558 0.016862407  0.4470    
9  hibdur.days          log_age  0.0013658595 0.009087186  0.8808    
10 hibdur.days        bm_before  0.0001200439 0.006859114  0.9861    
11 hibend.doy           log_age -0.4419838220 0.110796841  0.0001 ***
12 hibend.doy         bm_before  0.1114506430 0.083704034  0.1856    
13 hibend.doy   repro_activeyes -0.1698787534 0.199046222  0.3951    
14 hibend.doy         age.death  0.0286848754 0.100174816  0.7751    
15 repro_active         log_age  0.8731091300 0.271217194  0.0013  **
16 repro_active       age.death  0.4570170833 0.275831629  0.0975    

Signif. codes:  0 ‘***’ 0.001 ‘**’ 0.01 ‘*’ 0.05 ‘.’ 0.1 ‘ ’ 1
```

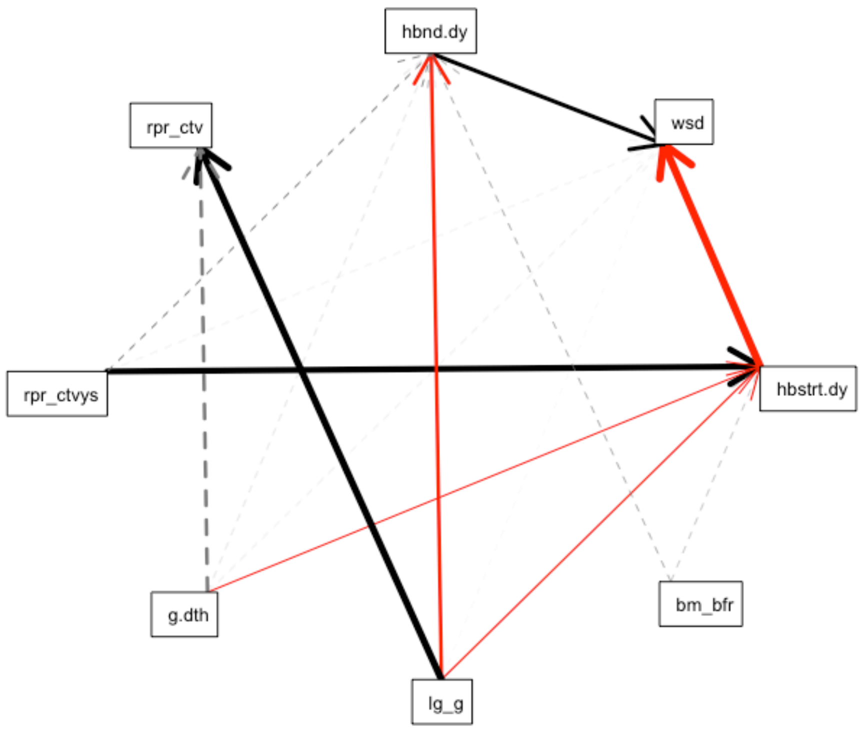

Fig. S1.1: Results plot of the path analysis for male edible dormice R-package piecewiseSEM[1]. Arrows represent unidirectional relationships among variables. Red lines indicate negative effects, black line positive effects. Solid lines indicate significant relationships, dashed lines non-significant relationships. The thickness of the line represents the strength of the effect, i.e., it is proportional to the absolute value of the scaled coefficient (see coef.table, sem.coefs for males [1]).

Females:

```
modlist=list(
lme(hibstart.doy~repro_active+age.death+log_age+bm_before,random=~1|year/diet/a.name,na.action
=na.omit,data=datf),
lme(hibdur.days~repro_active+hibend.doy+hibstart.doy+age.death+log_age+bm_before,random=~1|yea
r/diet/a.name,na.action=na.omit,data=datf),
lme(hibend.doy~log_age+age.death+repro_active+bm_before,random=~1|year/diet/a.name,na.action=n
a.omit,data=datf),
glmer(repro_active~log_age+age.death+(1|a.name)+(1|diet),family=binomial,data=datf)
)

> sem.fit(modlist,datf, .progressBar = FALSE,conditional=T)

$missing.paths

missing.path                         estimate std.error df crit.value p.value

1 repro_active ~ bm_before + age.death + log_age
                                    -0.0024    0.0115   NA  -0.2087  0.8347

2 hibend.doy ~ hibstart.doy + age.death + log_age + bm_before + repro_active
                                     0.0754    0.0716   84   1.0540  0.2949

$Fisher.C
  fisher.c df p.value
1      2.8  4   0.591

$AIC
   AIC    AICc  K   n
1 70.8 104.321 34 106

> sem.model.fits (modlist)
     Class   Family     Link   n   Marginal Conditional
1      lme gaussian identity 106 0.06575485   0.9990519 (model hibstart)
2      lme gaussian identity 106 0.88721109   0.9999031 (model hibdur.days)
3      lme gaussian identity 106 0.23722244   0.9375330 (model hibend)
4 glmerMod binomial    logit 106 0.21011177   0.4109037 (model repro)

> coef.table = sem.coefs(modlist, datf)
> coef.table
       response       predictor     estimate   std.error p.value
1  hibstart.doy repro_activeyes  15.66018241  7.87237108  0.0499   *
2  hibstart.doy         log_age -16.17922505 14.53748679  0.2689
3  hibstart.doy       age.death  -1.57683432  1.43441286  0.2747
4  hibstart.doy       bm_before   0.09285748  0.11511078  0.4221
5  hibdur.days     hibstart.doy  -0.93165812  0.03823779  0.0000 ***
6  hibdur.days       hibend.doy   0.92803146  0.05314982  0.0000 ***
7  hibdur.days        bm_before   0.09772778  0.04498413  0.0327   *
8  hibdur.days        age.death  -0.75925928  0.55768740  0.1771
9  hibdur.days  repro_activeyes  -1.67043213  3.09172169  0.5904
10 hibdur.days          log_age   1.31788831  6.03808858  0.8278
11 hibend.doy           log_age -45.04480313 10.45508755  0.0000 ***
12 hibend.doy         bm_before   0.16172194  0.08296344  0.0546
13 hibend.doy         age.death  -0.60513335  1.04100755  0.5626
14 hibend.doy   repro_activeyes   1.87171858  5.69266025  0.7431
15 repro_active         log_age   4.40659077  1.61300025  0.0063  **
16 repro_active       age.death  -0.14063443  0.18182462  0.4392

> sem.coefs(modlist,datf,standardize = "scale")
       response       predictor     estimate  std.error p.value
1  hibstart.doy repro_activeyes  0.463890414 0.23319807  0.0499   *
2  hibstart.doy         log_age -0.134288033 0.12066162  0.2689
3  hibstart.doy       age.death -0.120570783 0.10968085  0.2747
4  hibstart.doy       bm_before  0.074727542 0.09263629  0.4221
5  hibdur.days     hibstart.doy -0.825852141 0.03389524  0.0000 ***
6  hibdur.days       hibend.doy  0.599988662 0.03436230  0.0000 ***
7  hibdur.days        bm_before  0.069715687 0.03209005  0.0327   *
```

```
8   hibdur.days        age.death -0.051462817 0.03780013  0.1771
9   hibdur.days  repro_activeyes -0.043862615 0.08118309  0.5904
10  hibdur.days          log_age  0.009696395 0.04442479  0.8278
11  hibend.doy           log_age -0.512613387 0.11897971  0.0000 ***
12  hibend.doy         bm_before  0.178443027 0.09154136  0.0546
13  hibend.doy         age.death -0.063441469 0.10913801  0.5626
14  hibend.doy   repro_activeyes  0.076019572 0.23120655  0.7431
15  repro_active         log_age  1.234694188 0.45195671  0.0063  **
16  repro_active       age.death -0.363006480 0.46934478  0.4393

Signif. codes:  0 ‘***’ 0.001 ‘**’ 0.01 ‘*’ 0.05 ‘.’ 0.1 ‘ ’ 1
```

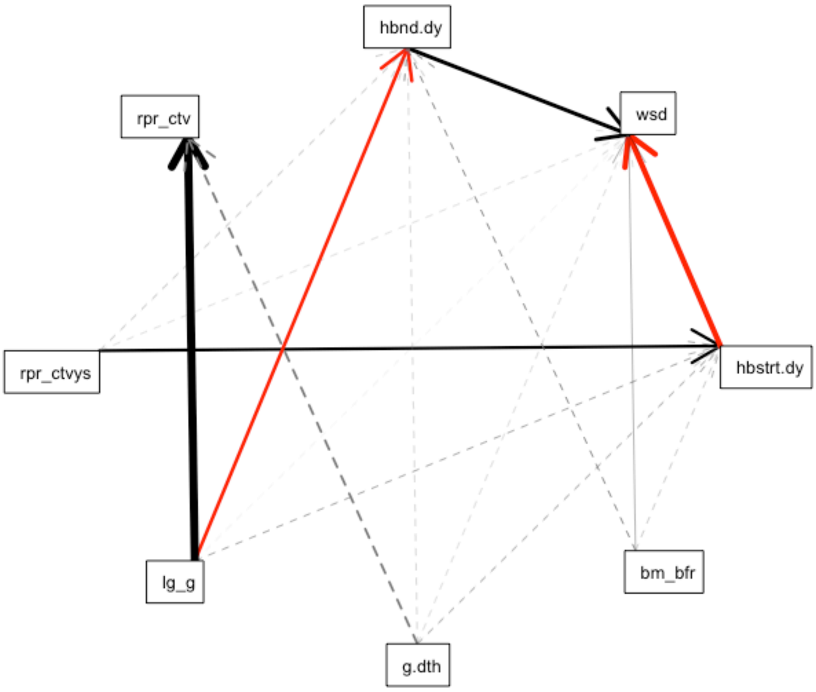

Fig. S1.2: Results plot of the path analysis for female edible dormice R-package piecewiseSEM[1]. Arrows represent unidirectional relationships among variables. Red lines indicate negative impacts, black line positive impacts. Solid lines indicate significant relations, dashed lines non-significant relations. The thickness of the line represents the strength of the effect i.e., it is proportional to the absolute value of the scaled coefficient (see coef.table, sem.coefs for females).

S2) Predictions from lm and lme models for effects of age (logarithmic scale) on reproduction and timing of hibernation

Table S2.1: Model prediction from our models included in the path analysis (see above, point S1) for logarithmic effects of age (in years) on the probability to develop tangible testes or give birth to a litter (reproduction), onset of hibernation (hibstart at day of year), hibernation duration (hibdur, number of days), and end of hibernation (hibend at day of year) for males and females. The age is given in years. Model predictions for timing of hibernation show pure age effects as investment in to reproduction was kept constant (at its mean).
Significant effects are indicated by an asterisk (*).

**males**				
age	reproduction*	hibstart*	hibdur	hibend*
1	0.31	248.99	255.65	141.80
2	0.50	239.74	255.71	130.99
3	0.62	234.33	255.75	124.66
4	0.69	230.49	255.78	120.17
5	0.74	227.51	255.80	116.69
6	0.77	225.08	255.82	113.84
7	0.80	223.02	255.83	111.44
8	0.82	221.24	255.84	109.35
9	0.84	219.67	255.85	107.51
10	0.85	218.26	255.86	105.87
11	0.86	216.99	255.87	104.38
12	0.88	215.83	255.88	103.02
13	0.88	214.76	255.89	101.77
**females**				
age	reproduction*	hibstart	hibdur	hibend*
1	0.03	242.99	275.94	166.92
2	0.10	238.12	276.33	153.36
3	0.18	235.27	276.57	145.43
4	0.27	233.25	276.73	139.80
5	0.35	231.68	276.86	135.44
6	0.42	230.40	276.96	131.87
7	0.49	229.32	277.05	128.86
8	0.55	228.38	277.13	126.24
9	0.59	227.55	277.20	123.94
10	0.64	226.81	277.26	121.88
11	0.67	226.14	277.31	120.01
12	0.71	225.53	277.36	118.31
13	0.73	224.97	277.41	116.75

S3) Accuracy of hibernation duration estimates based on nest-box controls

To justify the assumption that the absence in nest-boxes reflected accurately the time spent in hibernation we analysed data from enclosure housed animals implanted with temperature loggers (iButtons, (Maxim Dallas, USA,[4], n = 33 data sets). Hibernation was defined as the period between the first and last occurrence of body temperature < 25°C for at least 24h. We did not observe an influence of sex ($F_{1,30}$ = 1.34, P = 0.2561) or age ($F_{1,30}$ = 0.01, P = 0.9436) on the termination of hibernation between the two methods. The onset of hibernation was also not affected by age ($F_{1,30}$ = 0.83, P = 0.3689) or sex ($F_{1,30}$ = 0.43, P = 0.5181) between implanted or capture-recapture data sets.

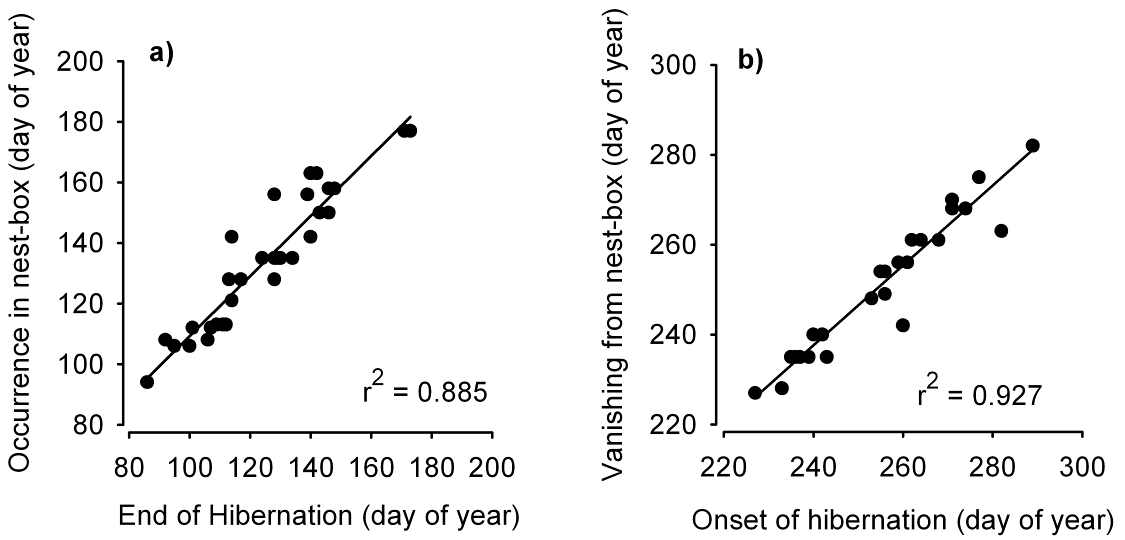

Fig. S3.1: Correlation between (a) occurrence in nest-boxes and termination of hibernation and (b) vanishing from nest-boxes and onset of hibernation.

S4) Relationships between variables of reproduction, hibernation and survival and age on a linear scale

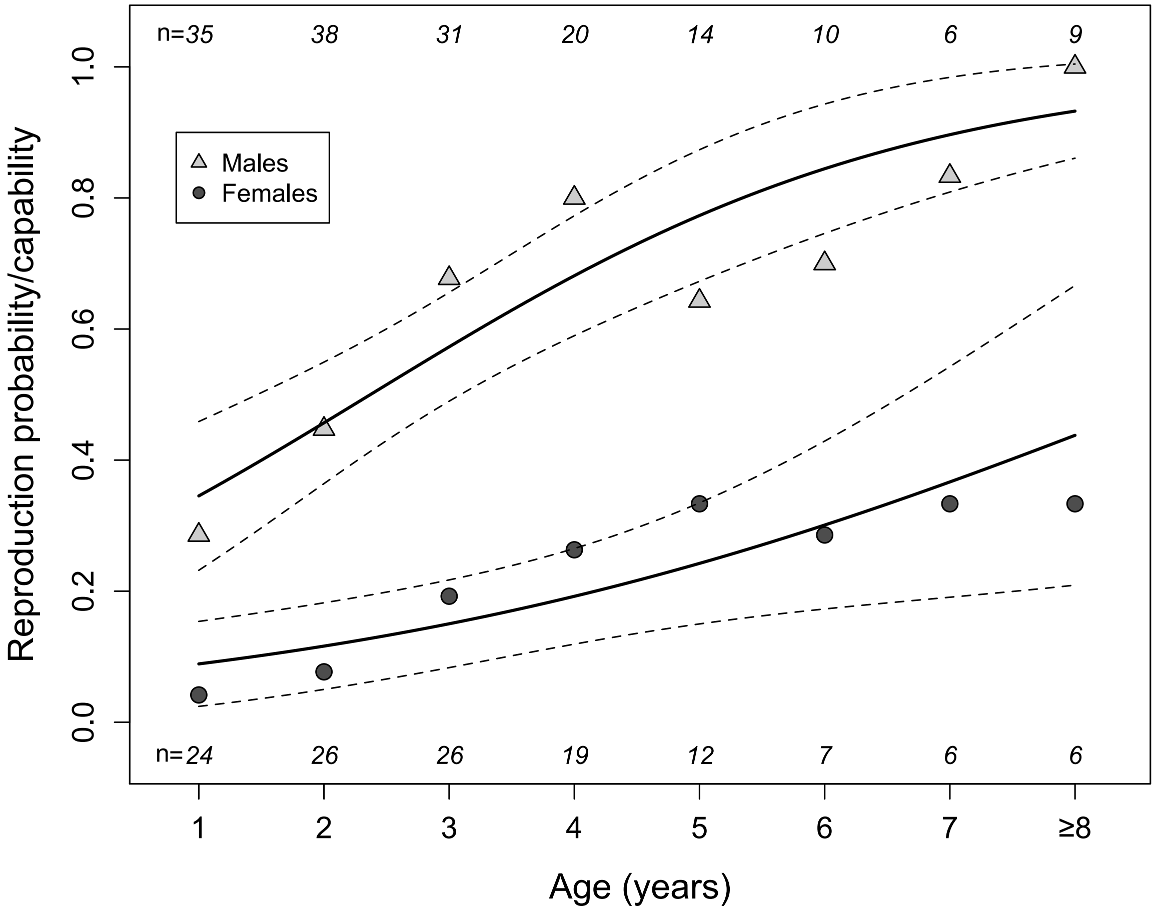

Fig. S4.1: Reproductive probability (females) or capability (males) as a function of age on a linear scale.

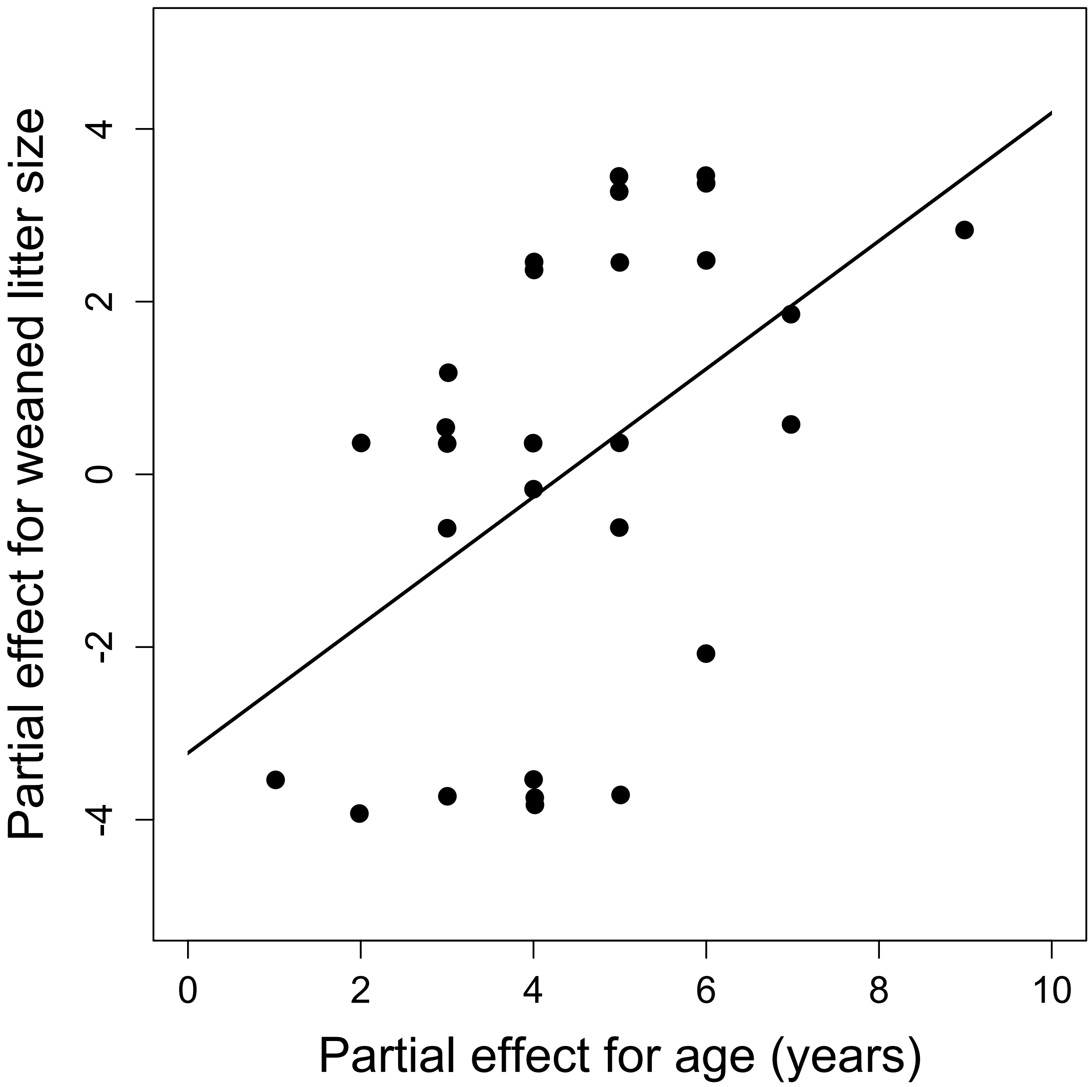

Fig. S4.2: Partial regression plot of linear effects of age on weaned litter size.

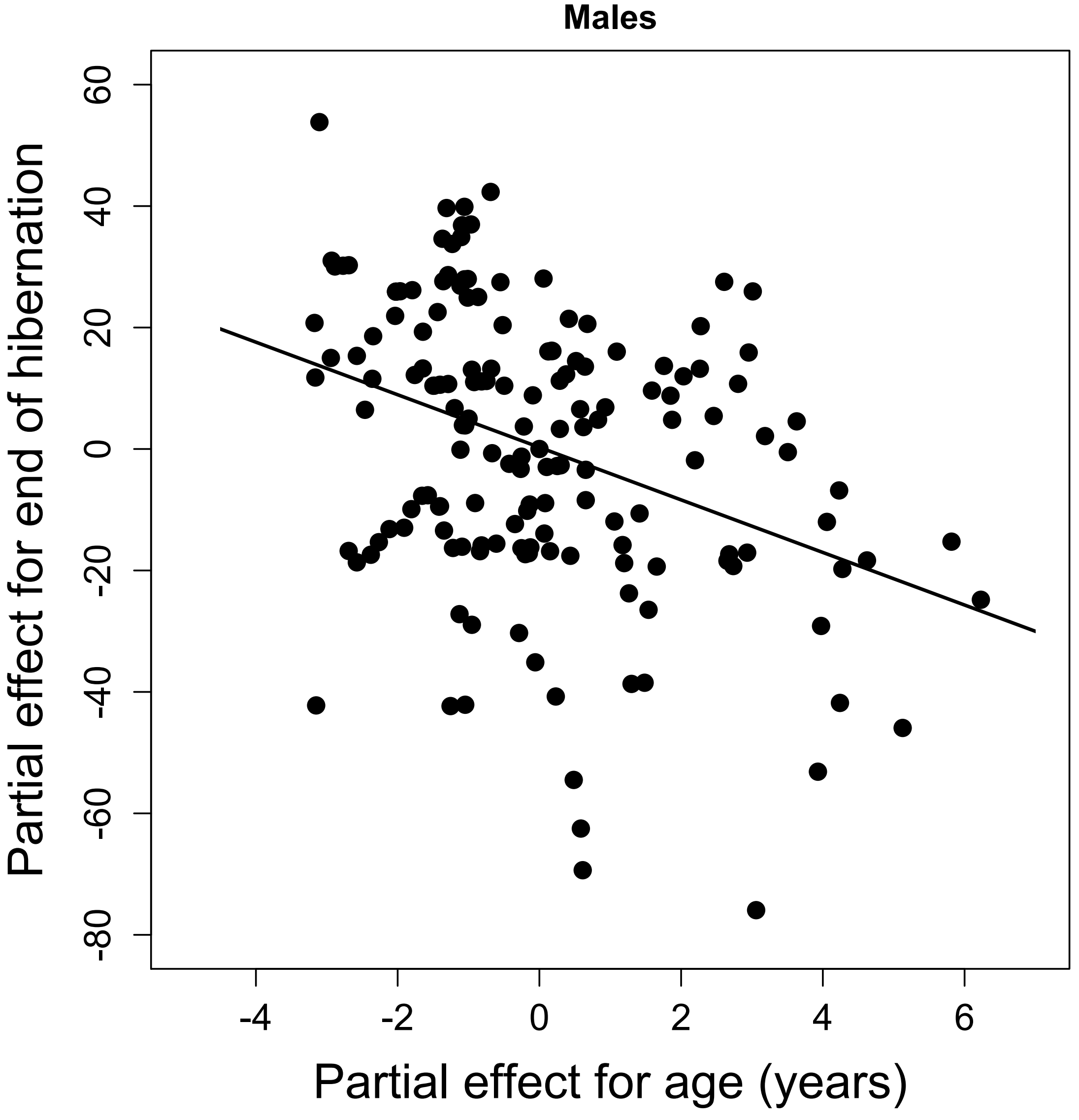

Fig. S4.3: Partial regression plot of linear effects of age on the time of emergence from hibernation in male dormice.

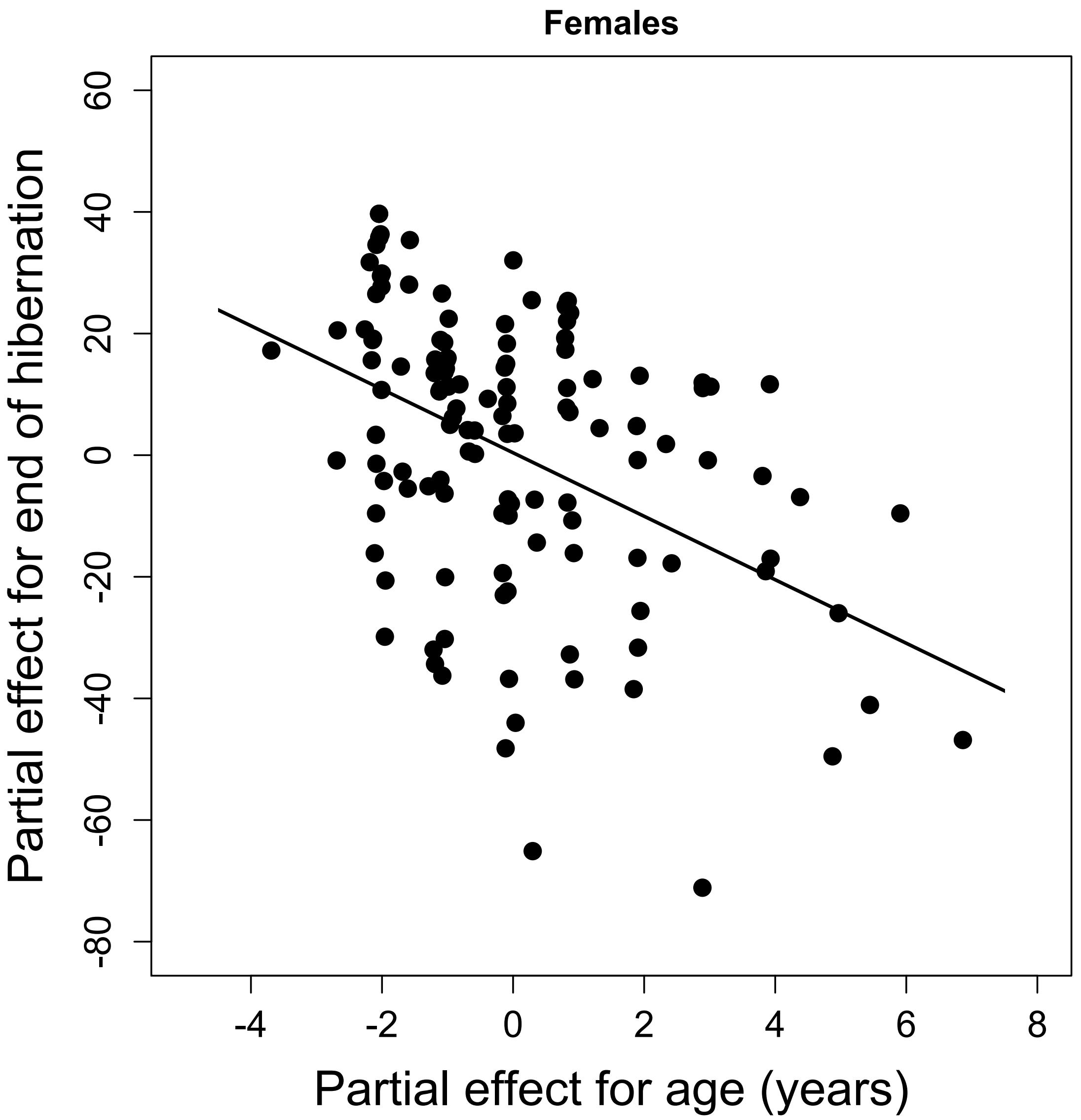

Fig. S4.4: Partial regression plot of linear effects of age on the time of emergence from hibernation in female dormice.

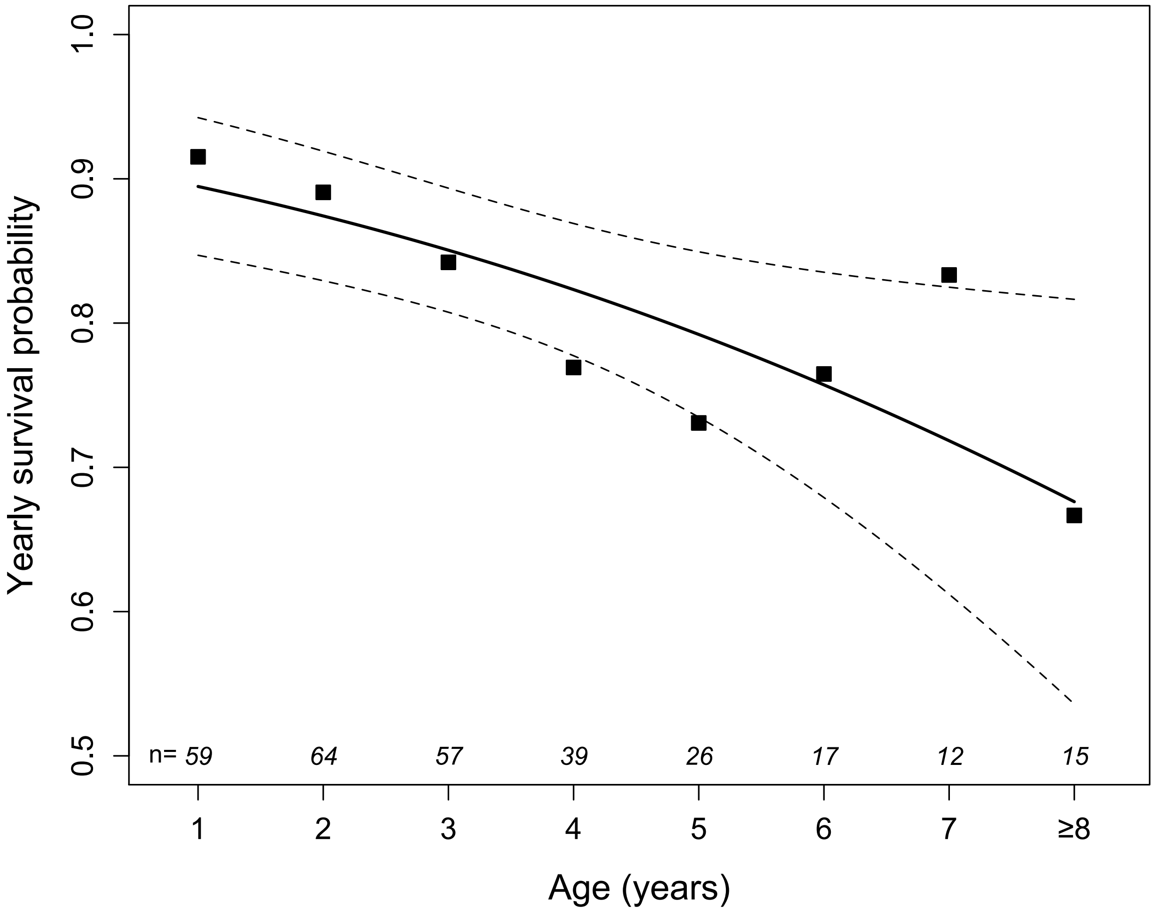

Fig. S4.5: Effects of age (on a linear scale) on yearly survival in dormice kept in outdoor enclosures.

References

1 Lefcheck, J. S. piecewiseSEM: Piecewise structural equation modelling in r for ecology, evolution, and systematics. *Methods Ecol. Evol.* **7**, 573-579, doi:10.1111/2041-210X.12512 (2016).

2 Pinheiro, J., Bates, D., DebRoy, S., Sarkar, D. & and the R Development Core team. nlme: Linear and Nonlinear Mixed Effects Models (R Package Version 3.1-127) (2016). <http://CRAN.R-project.org/package=nlme>.

3 Bates, D., Maechler, M., Bolker, B. & Walker, S. *lme4: Linear mixed-effects models using Eigen and S4. R package version 1.1-9*, <http://CRAN.R-project.org/package=lme4> (2015).

4 Bieber, C. & Ruf, T. Summer dormancy in edible dormice (*Glis glis*) without energetic constraints. *Naturwissenschaften* **96**, 165-171, doi:10.1007/s00114-008-0471-z (2009).
